# Supplementary material for: Relationship between Dietary Decanoic Acid and Coronary Artery Disease: A Population-Based Cross-Sectional Study
Source: Nutrients. 2023 Oct 10;15(20):4308. doi: 10.3390/nu15204308 (PMC10609701; doi:10.3390/nu15204308)
Supplement: Supplementary file 1 [file nutrients-15-04308-s001.zip › nutrients-2609364-supplementary.pdf]

Table S1. Distribution of variables with missing data comparing the raw data with the data by multiple imputation

	0	1	2	3	4	5	P value
Age, years	48.02 ± 16.83	48.02 ± 16.83	48.02 ± 16.83	48.02 ± 16.83	48.02 ± 16.83	48.02 ± 16.83	1.0000
Sex, %							1.0000
Male	47.94	47.94	47.94	47.94	47.94	47.94	
Female	52.06	52.06	52.06	52.06	52.06	52.06	
Race, %							1.0000
Mexican American	7.78	7.78	7.78	7.78	7.78	7.78	
Other Hispanic	5.04	5.04	5.04	5.04	5.04	5.04	
Non-Hispanic White	69.66	69.66	69.66	69.66	69.66	69.66	
Non-Hispanic Black	10.91	10.91	10.91	10.91	10.91	10.91	
Other race	6.61	6.61	6.61	6.61	6.61	6.61	
Education level, %							1.0000
<9th grade	5.04	5.04	5.04	5.04	5.04	5.04	
9–11th grade	10.14	10.14	10.14	10.14	10.14	10.14	
High school	23.70	23.70	23.70	23.70	23.70	23.70	
College	31.53	31.53	31.53	31.53	31.53	31.53	
Graduate or above	29.59	29.59	29.59	29.59	29.59	29.59	
PIR	3.05 ± 1.62	3.03 ± 1.75	3.03 ± 1.71	3.02 ± 1.72	3.03 ± 1.71	3.04 ± 1.78	0.2363
BMI, kg/m^2	29.02 ± 6.82	29.02 ± 6.82	29.03 ± 6.83	29.02 ± 6.82	29.02 ± 6.83	29.02 ± 6.82	1.0000
Hypertension, %							1.0000
No	62.11	62.11	62.11	62.11	62.11	62.11	
Yes	37.89	37.89	37.89	37.89	37.89	37.89	
Diabetes, %							1.0000
No	86.45	86.45	86.45	86.45	86.45	86.45	
Yes	13.55	13.55	13.55	13.55	13.55	13.55	
Smoking Status, %							1.0000
Never Smoking	54.60	54.60	54.60	54.60	54.60	54.60	
Quit Smoking	23.39	23.39	23.39	23.39	23.39	23.39	
Current Smoking	22.01	22.01	22.01	22.01	22.01	22.01	
Drinking status, %							1.0000
Never Drinking	19.90	19.90	19.90	19.90	19.90	19.90	
1-5 drinks/month	41.14	41.14	41.14	41.14	41.14	41.14	
5-10 drinks/month	7.58	7.58	7.58	7.58	7.58	7.58	
10+ drinks/month	14.05	14.05	14.05	14.05	14.05	14.05	
Unknown	17.33	17.33	17.33	17.33	17.33	17.33	
FBG, mmol/L	5.50 ± 1.79	5.49 ± 1.78	5.49 ± 1.78	5.49 ± 1.78	5.50 ± 1.78	5.49 ± 1.78	0.9992
HBA1c, %	5.60 ± 0.90	5.61 ± 0.90	5.61 ± 0.90	5.61 ± 0.90	5.61 ± 0.90	5.61 ± 0.90	0.9906
TC, mmol/L	5.07 ± 1.08	5.07 ± 1.08	5.07 ± 1.08	5.07 ± 1.09	5.07 ± 1.08	5.07 ± 1.08	0.9905
Triglyceride, mmol/L	1.71 ± 1.48	1.71 ± 1.47	1.71 ± 1.47	1.71 ± 1.47	1.71 ± 1.47	1.71 ± 1.47	0.9971

ALT, U/L	25.34 ± 19.79	25.22 ± 19.51	25.19 ± 19.50	25.23 ± 19.51	25.21 ± 19.50	25.19 ± 19.48	0.9377
BUN, mg/dL	4.85 ± 1.95	4.86 ± 1.95	4.87 ± 1.96	4.87 ± 1.96	4.87 ± 1.96	4.87 ± 1.96	0.8316
UA, umol/L	322.67 ± 83.09	323.12 ± 83.44	322.96 ± 83.48	322.66 ± 83.23	322.54 ± 83.31	322.63 ± 83.18	0.9394
SCR, umol/L	79.17 ± 31.74	79.16 ± 31.45	79.19 ± 31.44	79.18 ± 31.43	79.14 ± 31.44	79.21 ± 31.48	0.9997
eGFR, mL/min/1.73m2	91.81 ± 23.85	91.95 ± 24.31	92.03 ± 24.27	92.09 ± 24.33	92.05 ± 24.33	92.04 ± 24.40	0.6909
Energy, kcal	2099.99 ± 826.68	2099.99 ± 826.68	2099.99 ± 826.68	2099.99 ± 826.68	2099.99 ± 826.68	2099.99 ± 826.68	1.0000
DDA, g/d	0.48 ± 0.37	0.48 ± 0.37	0.48 ± 0.37	0.48 ± 0.37	0.48 ± 0.37	0.48 ± 0.37	1.0000
Protein, g/d	82.19 ± 34.87	82.19 ± 34.87	82.19 ± 34.87	82.19 ± 34.87	82.19 ± 34.87	82.19 ± 34.87	1.0000
Carbohydrate, g/d	250.88 ± 106.99	250.88 ± 106.99	250.88 ± 106.99	250.88 ± 106.99	250.88 ± 106.99	250.88 ± 106.99	1.0000
Fiber, g/d	16.83 ± 8.92	16.83 ± 8.92	16.83 ± 8.92	16.83 ± 8.92	16.83 ± 8.92	16.83 ± 8.92	1.0000
Total fat, g/d	81.10 ± 38.30	81.10 ± 38.30	81.10 ± 38.30	81.10 ± 38.30	81.10 ± 38.30	81.10 ± 38.30	1.0000
saturated fatty acids, g/d	26.52 ± 13.81	26.52 ± 13.81	26.52 ± 13.81	26.52 ± 13.81	26.52 ± 13.81	26.52 ± 13.81	1.0000
monounsaturated fatty acids, g/d	29.07 ± 14.52	29.07 ± 14.52	29.07 ± 14.52	29.07 ± 14.52	29.07 ± 14.52	29.07 ± 14.52	1.0000
polyunsaturated fatty acids, g/d	18.27 ± 9.86	18.27 ± 9.86	18.27 ± 9.86	18.27 ± 9.86	18.27 ± 9.86	18.27 ± 9.86	1.0000
Vitamin E, mg/d	8.32 ± 5.47	8.32 ± 5.47	8.32 ± 5.47	8.32 ± 5.47	8.32 ± 5.47	8.32 ± 5.47	1.0000
Vitamin K, μg/d	113.29 ± 216.73	113.29 ± 216.73	113.29 ± 216.73	113.29 ± 216.73	113.29 ± 216.73	113.29 ± 216.73	1.0000
Vitamin A, μg/d	646.30 ± 561.79	646.30 ± 561.79	646.30 ± 561.79	646.30 ± 561.79	646.30 ± 561.79	646.30 ± 561.79	1.0000
Vitamin B6, mg/d	2.09 ± 1.30	2.09 ± 1.30	2.09 ± 1.30	2.09 ± 1.30	2.09 ± 1.30	2.09 ± 1.30	1.0000
Vitamin C, mg/d	82.98 ± 76.74	82.98 ± 76.74	82.98 ± 76.74	82.98 ± 76.74	82.98 ± 76.74	82.98 ± 76.74	1.0000

Abbreviations: PIR Poverty income ratio, BMI body mass index, FPG fasting plasma glucose, HbA1c hemoglobin A1c, BUN Blood urea nitrogen, TC total cholesterol, UA uric acid, SCR Serum creatinine, eGFR estimated glomerular filtration rate, ALT Alanine aminotransferase, DDA Dietary decanoic acid.

* Data are presented as mean ± standard deviation and numbers (%) as appropriate.

Table S2. Relative odds of CAD according to DDA in different models among American adults based on multiple imputed data

Exposure	Coronary artery disease (OR (95% CI))									
	Model 1					Model 2				
	MI.1	MI.2	MI.3	MI.4	MI.5	MI.1	MI.2	MI.3	MI.4	MI.5
DDA	0.62 (0.55, 0.70)	0.62 (0.55, 0.70)	0.62 (0.55, 0.70)	0.62 (0.55, 0.70)	0.62 (0.55, 0.70)	0.76 (0.63, 0.91)	0.75 (0.63, 0.90)	0.76 (0.63, 0.91)	0.76 (0.63, 0.91)	0.76 (0.63, 0.91)
Lg DDA	0.71 (0.65, 0.77)	0.71 (0.65, 0.77)	0.71 (0.65, 0.77)	0.71 (0.65, 0.77)	0.71 (0.65, 0.77)	0.80 (0.71, 0.91)	0.80 (0.71, 0.91)	0.80 (0.71, 0.91)	0.80 (0.71, 0.91)	0.80 (0.71, 0.91)
DDA Tertile										
T1	1.0	1.0	1.0	1.0	1.0	1.0	1.0	1.0	1.0	1.0
T2	0.79 (0.72, 0.87)	0.79 (0.72, 0.87)	0.79 (0.72, 0.87)	0.79 (0.72, 0.87)	0.79 (0.72, 0.87)	0.87 (0.78, 0.96)	0.86 (0.77, 0.96)	0.87 (0.78, 0.97)	0.86 (0.78, 0.96)	0.87 (0.78, 0.96)
T3	0.70 (0.63, 0.77)	0.70 (0.63, 0.77)	0.70 (0.63, 0.77)	0.70 (0.63, 0.77)	0.70 (0.63, 0.77)	0.82 (0.72, 0.94)	0.81 (0.71, 0.93)	0.82 (0.72, 0.94)	0.82 (0.72, 0.94)	0.82 (0.72, 0.94)

Abbreviations: MI multiple imputed; Lg DDA value was log10-transformed. Model 1 adjusts for none; Model 2 adjusts for sex, age, race, education levels, PIR, BMI, hypertension, Diabetes, Smoking Status, Drinking Status, TC, triglyceride, BUN, UA, SCR, eGFR, ALT, Energy, Protein, Carbohydrate, Fiber, Total fat, and multivitamin.

Table S3. Pool estimates from multiple imputed data

Data										
Var.	beta 1	se 1	beta 2	se 2	beta 3	se 3	beta 4	se 4	beta 5	se 5
DDA	-0.274	0.094	-0.288	0.091	-0.274	0.094	-0.274	0.094	-0.274	0.094
lg DDA	-0.223	0.063	-0.223	0.063	-0.223	0.063	-0.223	0.063	-0.223	0.063
DDA Tertile										
T1	Refer	Refer	Refer	Refer	Refer	Refer	Refer	Refer	Refer	Refer
T2	-0.139	0.053	-0.151	0.056	-0.139	0.057	-0.151	0.053	-0.139	0.053
T3	-0.198	0.068	-0.211	0.069	-0.198	0.068	-0.198	0.068	-0.198	0.068
Pooled estimates										
Var.	Coefficients	Se	P value	OR	95%CI low	95%CI up				

DDA	-0.277	0.093	0.003	0.758	0.631	0.910
lg DDA	-0.223	0.063	<0.001	0.800	0.707	0.906
DDA Tertile						
T1	Refer	Refer	Refer	Refer	Refer	Refer
T2	-0.144	0.055	0.008	0.866	0.778	0.964
T3	-0.201	0.068	0.003	0.818	0.715	0.935

Abbreviations: Lg DDA value was log10-transformed. Adjusts for sex, age, race, education levels, PIR, BMI, hypertension, Diabetes, Smoking Status, Drinking Status, TC, triglyceride, BUN, UA, SCR, eGFR, ALT, Energy, Protein, Carbohydrate, Fiber, Total fat, and multivitamin.
